# Production of Ni_0.5_Co_0.5_Fe_2_O_4_/activated carbon@chitosan magnetic nanobiocomposite as a novel adsorbent of methylene blue in aqueous solutions

**DOI:** 10.1038/s41598-023-33470-y

**Published:** 2023-04-15

**Authors:** Zakaria Dastoom

**Affiliations:** Shahid Beheshti High School, Toyserkan, Hamedan Province Iran

**Keywords:** Environmental sciences, Natural hazards, Risk factors, Materials science, Nanoscience and technology

## Abstract

Methylene blue is a cationic dye, not degraded naturally due to its aromatic rings. Accordingly, biological, chemical, and physical water treatment methods have been proposed for its removal. Adsorption is an economical and effective method in this regard. In this study, the nickel–cobalt ferrite/activated carbon@chitosan magnetic nanobiocomposite was synthesized as an adsorbent. The nano-adsorbent was evaluated with FESEM, which estimated the particle size at ~ 16.64 nm. According to EDAX analysis, the purity of particles was 99%. XRD characterization showed the successful coverage of chitosan, correct placement of nickel–cobalt ferrite, and the nono-structure of crystallites. The specific surface area was 316 m^2^/g using the BET theory and 285 m^2^/g using the Langmuir theory, and the porosity volume was 0.18 cm^3^/g. According to the VSM analysis, magnetic reluctance and coercive force were 1.1 emu/g and 499 Oe, respectively. The FTIR analysis showed that the reaction was successful, and methylene blue was present on the adsorbent surface. The methylene blue adsorption test indicated that 388 mg/g of the dye was adsorbed (97% dye removal), and the final concentration reached 6 mg/L after 8 h. The point of zero charge (pHpzc) was 6.8.

## Introduction

Improper disposal of pollutants such as heavy metal ions, dyes, pharmaceutical effluents, pesticides, and organic compounds in aquatic environments is a global challenge^1^. Dyes are pollutants that can cause mutagenesis and carcinogenesis. They are used as base chemicals in various industries such as leather, paper, textile, rubber, plastic, drug, and cosmetics^1^. Disposal of dye-containing effluent into water sources increases water pollution, blocks the sunlight, and disturbs the ecological balance^2^. In addition, aromatic rings in the structure of some anionic and cationic dyes turn them toxic and result in dizziness, jaundice, cyanosis, burning, allergy, vomiting, and diarrhea if degraded in the body^2^. As a result, the removal of these pollutants from water is necessary. As technology develops, new methods have been introduced for water treatment. Water treatment methods are divided into three groups: chemical, including oxidation^3^, ion exchange^4^, and precipitation^5^; physical, including filtration^6^, adsorption^7^, air flotation^8^, and coagulation^9^; and biological, including aerobic and anaerobic^2^. In general, due to the adsorption's low cost and high efficiency, it is the most proper and effective water treatment method. Various compounds have been used as an adsorbent, e.g., carbon nanotubes^10^, activated carbon^11^, zeolite^12^, metal oxides^13^, chitosan^14^, core–shell nanomaterials^15^, magnetic nanocomposites^16^, silicone^17^, and bilayer hydroxides^18^. Among magnetic composites, cobalt ferrite/montmorillonite^19^ and graphene oxide/chitosan^20^ were evaluated for the removal of methylene blue. However, most of these compounds lack porous structure, high chemical stability, biological structure, and easy isolation properties at a time. For example, chitosan cannot be isolated easily. This study aims to synthesize Ni_0.5_Co_0.5_Fe_2_O_4_/Activated carbon@Chitosan as a nanobiocompo-site with all favorable features of a nano-absorbent. Activated carbon has a porous structure and is a bio-friendly and chemically stable compound that was used in this nano-absorbent. In addition, chitosan is a natural polymeric adsorbent able to adsorb dyes due to the hydroxyl and amine groups in its polymeric chains^21^. Chitosan was used for its biological properties, preventing dispersion of nanobiocomposite in water, proper reaction with dyes, and improving the adsorption process. Finally, nickel–cobalt ferrite was used to provide the magnetic separation of nanocomposite from aqueous solutions. BET analysis was performed for evaluating porosity, FESEM for confirming the intended morphology, XRD for confirming the crystallization properties and evaluating the crystallites size, FTIR for confirming the reaction success and the presence of methylene blue after adsorption of the dye by the nanocomposite, VSM for evaluating the nanoparticles magnetic properties, and EDAX for assessing the compound purity. The methylene blue adsorption test was performed to optimize parameters effective on the adsorption process, such as adsorption dose, methylene blue initial concentration, pH, and temperature. PHpzc was also obtained to evaluate the effect of pH on the adsorption process in terms of surface charge.

## Materials and methods

### Materials

FeCl_3_·6H_2_O, CoCl_2_·6H_2_O, NiCl_2_·6H_2_O, FeCl_2_·4H_2_O, and NaOH were purchased from the Merck Company, and chitosan from the Sigma Company. In this study, we used activated carbon made in Tuyserkan, Iran, and deionized water.

### Synthesis of Ni_0.5_Co_0.5_Fe_2_O_4_@AC/Ch magnetic nanobiocomposite

A homogenous solution of iron salt was made by adding 0.6 g of FeCl_2_·4H_2_O and 1.2 g of FeCl_3_·6H_2_O to 100 mL of deionized water. Then 0.45 g of NiCl_2_·6H_2_O and 0.45 g CoCl_2_·6H_2_O were added to the reaction container and left until homogenization. After a sufficient period, 1 g of activated carbon was added to the container and the solution was stirred for 30 min. Then, 100 mL of 1 M sodium hydroxide was dropped for 1 h to terminate the reaction. The obtained powder was extracted by an external field and dried at 100 °C for 24 h. Finally, the product was functionalized with 1 g of chitosan.

### Characterization

We evaluated the microstructure, morphology, and chemical compound of the nanocomposite with a Field Emission Scanning Electron Microscope with Energy Dispersive X-Ray Spectroscopy (FE-SEM- EDAX) (Zeiss Sigma 300). The X-Ray Diffraction (XRD) patterns at angles 2ϴ = 10–80 were used to identify the crystallography of the nanoparticle using a copper filter. We evaluated the bonding of the nanobiocomposite and confirmed the adsorption of methylene blue on the nanocomposite surface using Fourier transform infrared spectroscopy (FT-IR) with a Rayleigh-WQF-10 instrument in the range of 450–4000 cm/1. The adsorption and desorption isotherm and the specific surface area were also investigated using the Brunauer–Emmett–Teller (BET) and Langmuir theories.

### Adsorption test

To evaluate the effect of time on the process of dye absorption by nanoabsorbent, 250 ml of methylene blue with a concentration of 200 mg/liter was prepared and divided into four solutions, one solution was used as a control and 0.1 g of nanoabsorbent was added to each of the other solutions. UV–VIS spectra of three solutions were prepared after 2, 4 and 8 h and the final concentration of the solution was calculated after 8 h. The amount of removal after eight hours was obtained from Eq. (1):

1 $$R = \frac{{\left( {C_{0} - C_{t} } \right)*100}}{{{\text{C}}_{0} }}$$

In the above equation, where C_t_ and C_0_ are the initial concentration and the concentration at time t, respectively, in mg/L. The amount of dye absorbed by the absorber is obtained from Eq. (2):

2 $${\text{qe}} = \frac{{{ }\left( {{\text{C}}_{{\text{t}}} - {\text{C}}_{0} } \right){\text{*m}}}}{{\text{V}}}$$

where C_t_, C_0_ are respectively the initial concentration and concentration at time t in mg/liter, m is the mass of adsorbent in grams and V is the solution volume in liters.

## Results and discussion

### Characterization of the magnetic nanobiocomposite

#### FTIR spectrum of nanobiocomposite before and after absorption of methylene blue

**Figure 1:**
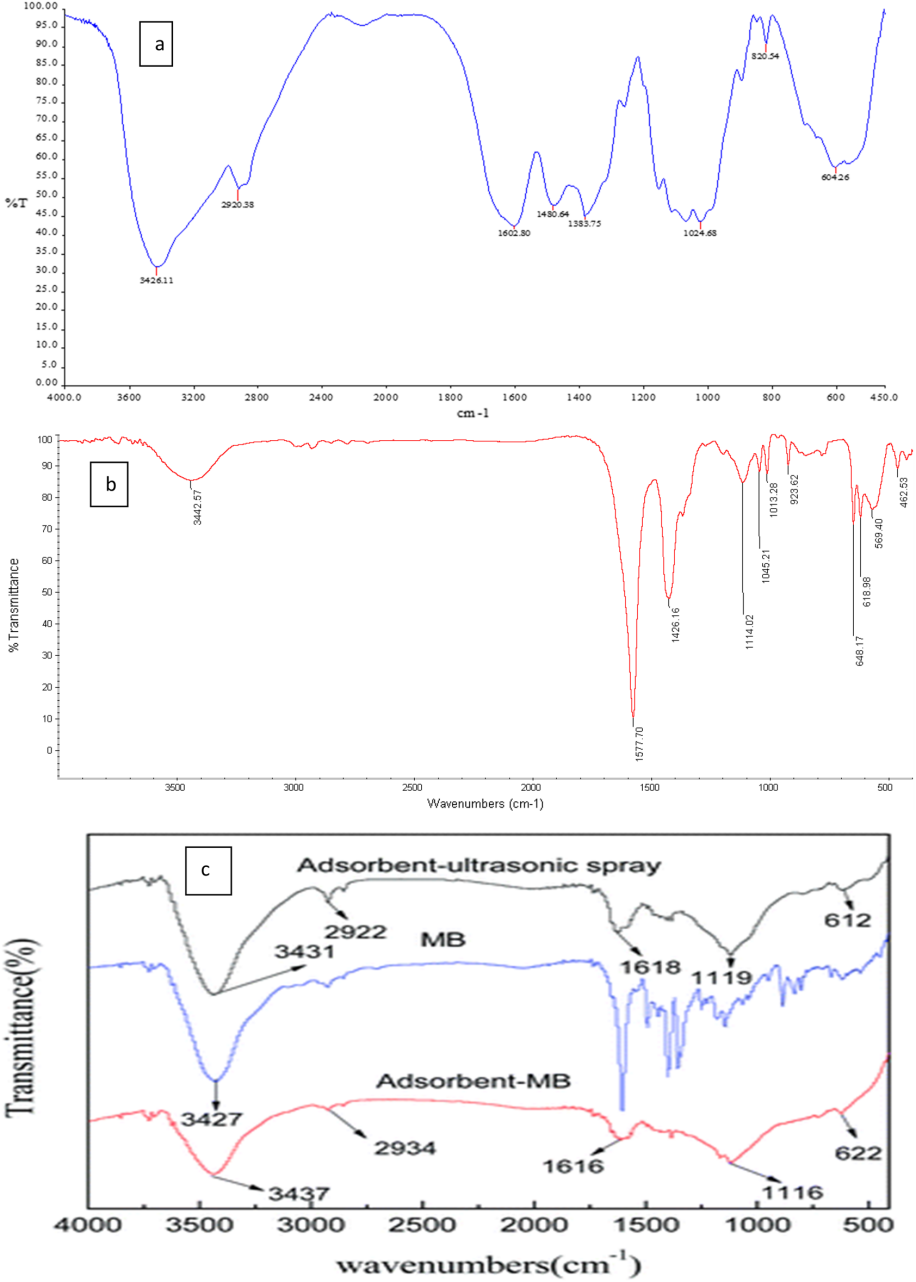
The FT-IR spectrum of the Ni_0.5_Co_0.5_Fe_2_O_4_@AC/Ch nanobiocomposite (**a**) Before adsorption (**b**) after adsorption (**c**) The FT-IR spectrum of the Methylene blue^22^.

The FTIR spectrum of the nano adsorbent before and after the adsorption process is shown in Fig. 1a,b, respectively, and the methylene blue spectrum is shown in Fig. 1c^22^. The FTIR spectrum of magnetic nanobiocomposite Ni_0.5_Co_0.5_Fe_2_O_4_/AC@Ch was studied at 450–4000 cm^−1^ before methylene blue absorption. The peaks were seen at 3426 cm^−1^, 2920 cm^−1^, 1603 cm^−1^, 1384 cm^−1^, 1025 cm^−1^, 821 cm^−1^, 604 cm^−1^. The peak of 3426 cm^−1^ was due to the stretching vibration of O–H and N–H bonds in the chitosan^23^. The peaks 2920 cm^−1^ and 1384 cm^−1^ pertained to the stretching vibration of C–H in the CH2, CH, and CHOH bonds, respectively^16,24^. The peak 1603 cm^−1^ occurred due to the stretching vibration of C=O in the NH=C=O chain^25^. The present peak at 1025 cm^−1^ indicates the asymmetric stretching of the C–O–C bond^26^. The peak 821 cm^−1^ was due to the bending vibration of C=C and the peak 604 cm^−1^ pertained to the stretching vibration of Ni–O, Fe–O, and Co–O in the tetrahedral or octahedral inverse spinel structure. by checking Fig. 1b,c and comparing the spectrum of methylene blue and nanoabsorbent after adsorption procss, we understand that the peak of nanoabsorbent spectrum at 3442 cm^−1^ is related to OH absorbed in water or N–H in methylene blue, Because the peak is wider compared to the state before methylene blue absorption and is more related to the 3427 cm^−1^ peak in pure methylene blue spectrum. The intense peak of 1578 cm^−1^ is related to the stretching vibration of the C=O bond. The intense peak of 1426 cm^−1^ is related to C–H stretching vibration in bonds such as CH3 in methylene blue because it is more intense than the peak related to C–H stretching vibration. The peak at 1114 cm^−1^ corresponds to the C–N bond in methylene blue. The rest of the peaks in Fig. 1(c) are also related to the nanobiocomposite structure, the three mentioned peaks 1578 cm^−1^, 1426 cm^−1^, 1114 cm^−1^ well confirm the presence of methylene blue after the absorption process on the surface of the nanobiocomposite.

#### FESEM and element EDAX analyses of the magnetic nanobiocomposite

**Figure 2:**
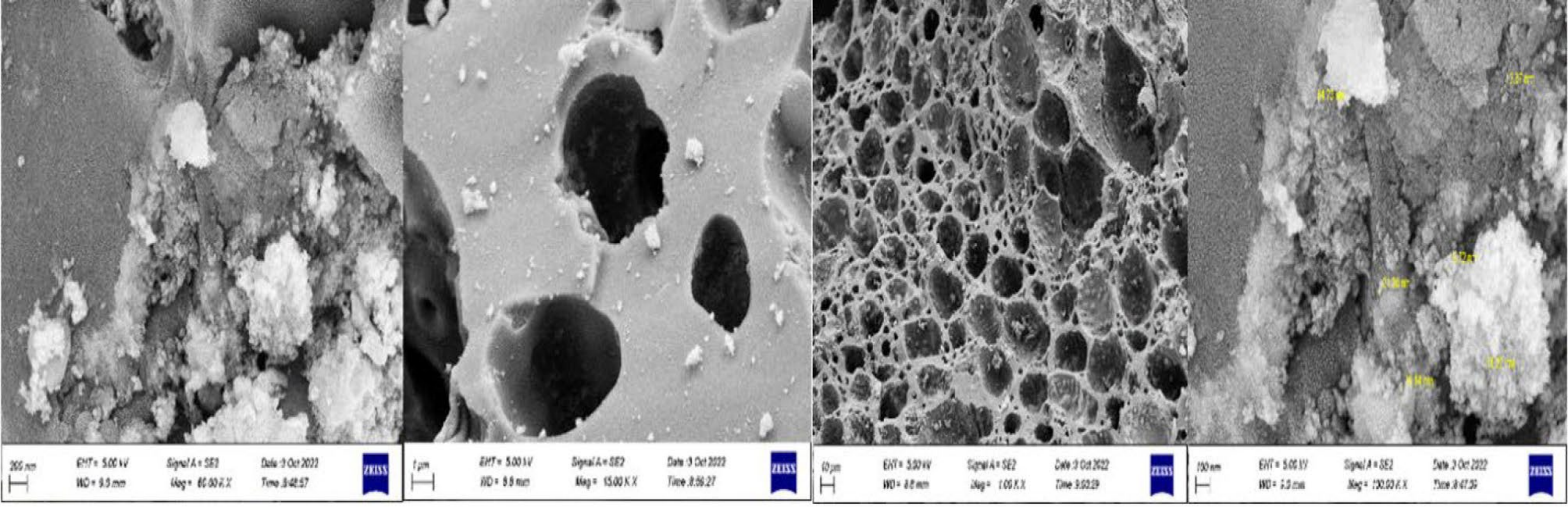
FESEM of the Ni_0.5_Co_0.5_Fe_2_O_4_@AC/Ch magnetic nanobiocomposite.

**Figure 3:**
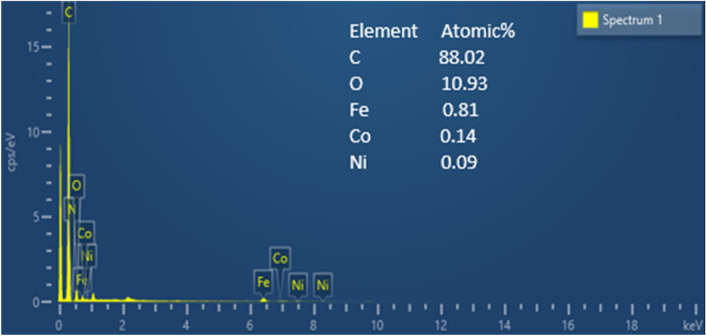
Elemental EDAX analysis of the Ni_0.5_Co_0.5_Fe_2_O_4_@AC/Ch magnetic nanobiocomposite.

Figure 2 represents the FESEM of the magnetic nanobiocomposite at 100, 200, 1000, and 10,000 nm magnifications. According to these images, the particle sizes are about 16.64 nm, and the ferrite nickel–cobalt particles are present as small bulks on the activated carbon surface. Element EDAX analysis is shown in Fig. 3, according to which, the ratios of Fe, Co, Ni, C, and O are consistent with the used ratios, indicating the high purity of 99% the of nanobiocompo-site. These two analyzes show the effect of the use of stabilizers and the quality of the raw materials.

### Magnetic properties of the nanobiocomposite

**Figure 4:**
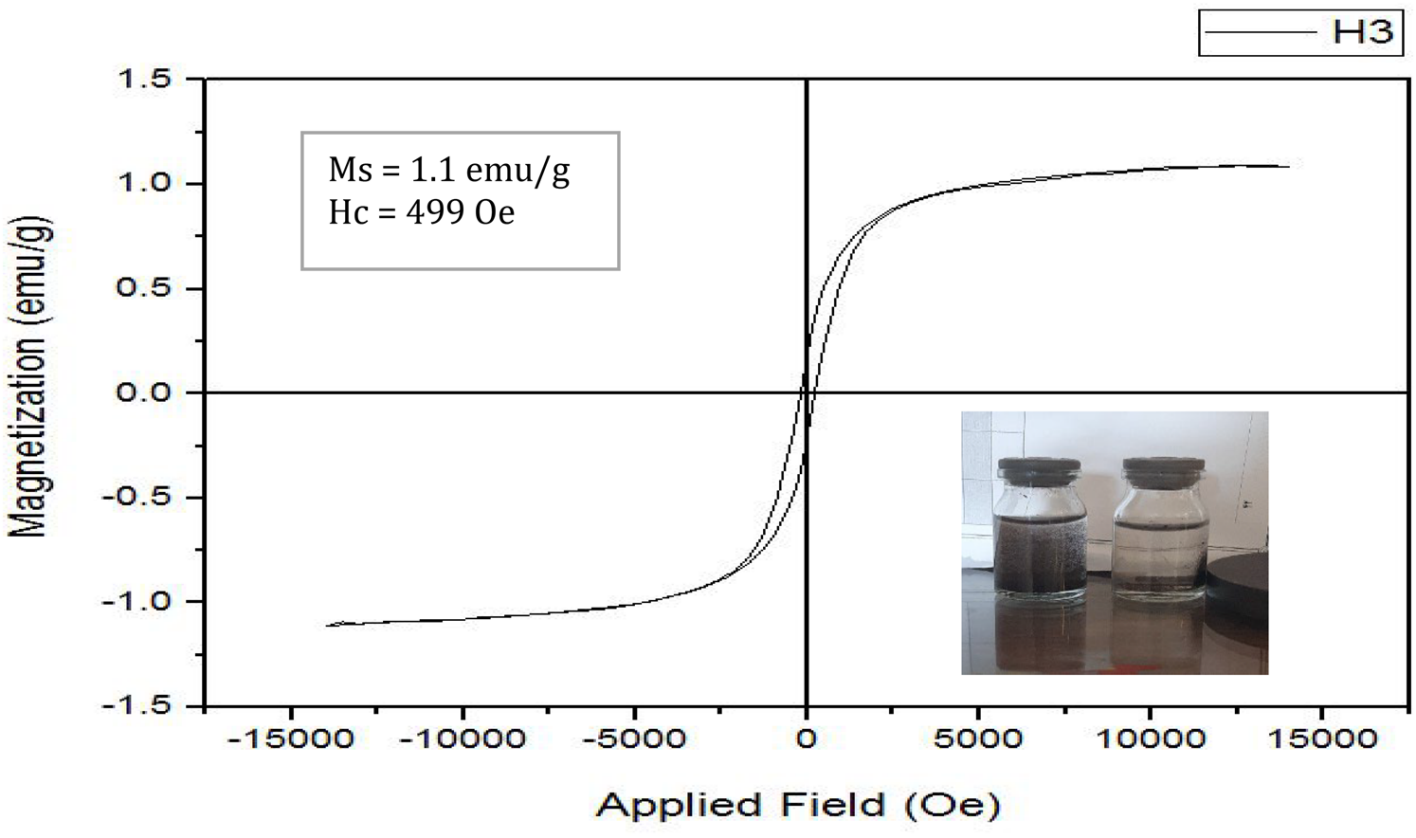
The magnetization curve (VSM) of the Ni_0.5_Co_0.5_Fe_2_O_4_@AC/Ch nanobiocomposite and its easy desorption from aqueous solutions by an external field.

Figure 4 represents the magnetization curve of Ni_0.5_Co_0.5_Fe_2_O_4_@AC/Ch. According to this curve, the compound is a ferromagnetic nanoadsorbent with a coercive force (H_c_) of 499 Oe and saturation magnetization (M_s_) of 1.1 emu/g. These values indicate the efficiency of magnetic separation of the nanoadsorbent. As shown in Fig. 4, the nanoadsorbent was homogenously dispersed in the aqueous solution and easily desorbed after applying an external field. Since this nanobiocomposite uses the magnetic separation method, it is cost-effective and reduces the time of separation.

### X-ray diffraction (XRD) patterns of the magnetic nanobiocomposite

**Figure 5:**
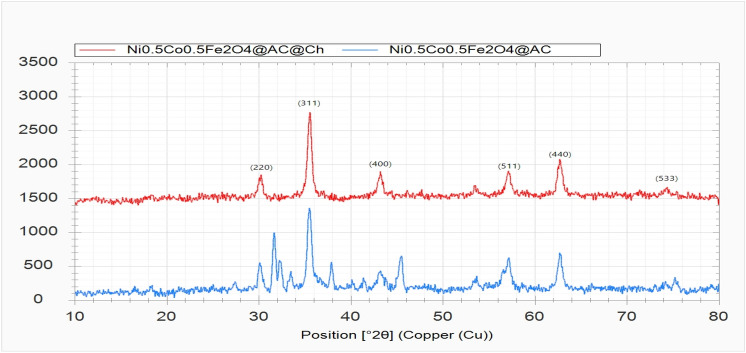
Comparison of X-ray diffraction patterns of Ni_0.5_Co_0.5_Fe_2_O_4_/AC and Ni_0.5_Co_0.5_Fe_2_O_4_/AC@Ch.

The X-ray diffraction patterns of Ni_0.5_Co_0.5_Fe_2_O_4_@AC/Ch are shown in Fig. 5. Peaks were observed at 2ϴ of 30.3° (220), 35.6° (311), 43.2° (400), 57.26° (511), 62.68° (440), and 74.4° (533). These peaks indicate the cubic spinel structure of ferrite nickel–cobalt, and their similarity with the patterns of Chitosan, Fe3O4, and Co3O4 shows that the present study is consistent with the research literature. The size of crystallites was estimated at 96 nm using XPert HighScore Plus software. In the X-ray diffraction pattern of Ni_0.5_Co_0.5_Fe_2_O_4_/AC, the peaks were observed at about 18.42°, 27.46°, 30.14°, 31.7°, 32.3°, 33.48°, 35.48°, 37.88°, 45.52°, 57.16°, and 62.68°. A comparison of these two diffraction patterns shows that adding chitosan resulted in the filling of inter-crystal planes in the nanocomposite, severely reduced the peaks, and inclined them to higher degrees. Also, the peaks indicate the proper placement of nickel–cobalt ferrite.

### BET analysis of the magnetic nanobiocomposite

**Figure 6:**
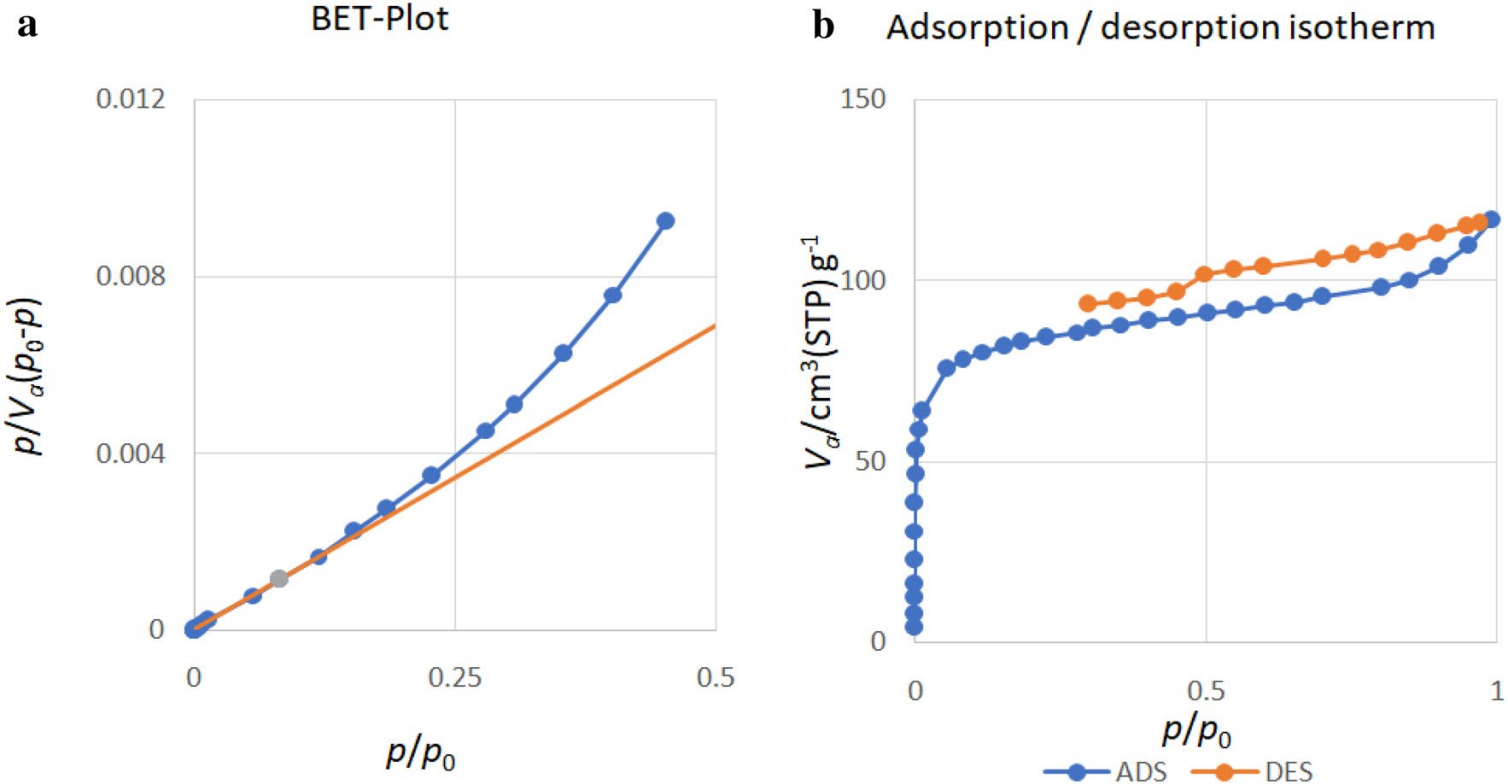
(**a**) The magnetic nanobiocomposite adsorption/desorption cure, (**b**) the magnetic nanobiocomposite BET curve.

The BET surface area was obtained using the adsorption/desorption diagram in Fig. 6. The BET equation was used to calculate the volume of the absorbed monolayer, from which the surface area of the absorber is calculated^3^. The surface area of Ni_0.5_Co_0.5_Fe_2_O_4_ nanobiocomposite /AC@Ch based on Brunner-Emmett-Thaler theory, the value of 316.23 m^2^/g was obtained from Eq. (3):

3 $$S = \frac{{{\text{VmNa}}}}{{22400\;{\text{m}}}}$$

where S is the surface area of the material, Na is Avogadro's number, m is the mass of the tested sample in grams, 22,400 is the volume occupied by one mole of absorbed gas in the standard state, and Vm is the volume of absorbed gas, which is obtained from Eq. (4):

4 $$Vm = \frac{1}{{{\text{A}} + 1}}$$

In the equation above, Vm is the volume of absorbed gas, A is the slope of the BET diagram, the value of the specific surface through Langmuir's theorem based on five assumptions that include ((1) a completely homogeneous surface and there is no priority between molecular adsorption sites. (2) Each adsorption site has only one absorb the molecule and always a single layer of molecules are absorbed on the surface. (3) The absorption mechanism is the same on the surface of all molecules. (4) There is no interaction between gas molecules. (5) Speed absorption and desorption are equal.) is established, with a value of 286.55 m^2^ g^−1^. Both of these theories confirm the use of this material as a suitable surface adsorbent by showing a high specific surface value. The total defect volume was also 0.18 cm/g (p/p_0_ = 0.990), which is a relatively high value. The adsorption isotherms are classified based on the strength of the interaction between the sample surface and the adsorbent surface and the existence or absence of pores. The nanobiocomposite adsorption isotherm was of type IV which is characteristic of mesoporous material. In addition, the adsorption/desorption hysteresis in the diagram shows the conical geometry of the pores. Table 1 represents the comparison between the surface area of adsorbents in previous literature and the present study. As shown in this table, the specific surface of Ni_0.5_Co_0.5_Fe_2_O_4_/AC@Ch is higher than most organic and non-organic composites. The relatively larger specific surface areas and total pore volumes of the magnetic nanobiocomposite confirm its dye removal capability.

**Table 1:** Comparison of the adsorbents surface area (S_BET_) in pervious literature and the present study.

Adsorbent	S_BET_ (m^2^/g)	References
Ni_0.5_Co_0.5_Fe_2_O_4_/AC@Ch	316.23	This study
CoFe_2_O_4_/AC@Ch	474.36	^26^
Fe_3_O_4_@SiO_2_ Core–Shell	300.80	^15^
Activated carbon/NiFe_2_O_4_	157.10	^27^
Fe_3_O_4_-GS	62.43	^28^
CTS-MNPs	112	^29^
Cs-m-GMCNTs	39.20	^21^
CoFe_2_O_4_-chit	2	^1^

### PHpzc

**Figure 7:**
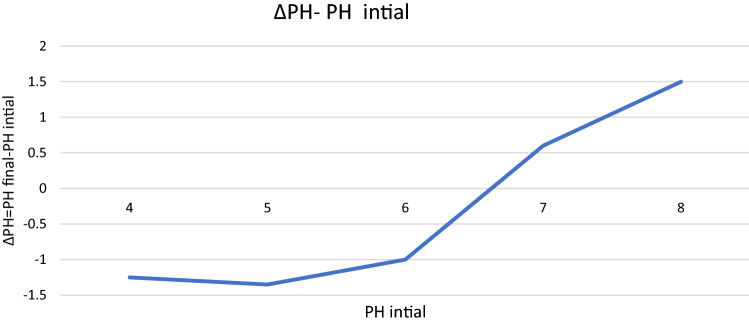
∆PH–PH intial diagram of magnetic nanobiocomposite.

The value of PHpzc was obtained by solid addition method of 6.8. That is, at a pH less than 6.8 bar, the absorbent surface is positive and at a pH greater than 6.8 bar, the absorbent surface is negative. Given that we know that methylene blue is a cationic dye, it is natural that its absorption rate is not as high as that of anionic dyes. Because at a lower pH of 6.8, methylene blue and the adsorbent both have a positive charge, so they repel each other, so in this condition, the absorption rate of methylene blue is lower than that of anionic dyes. But at pH higher than 6.8, the absorption rate of methylene blue is not high compared to the ideal case of anionic dyes, because in this case the number of OH– increases. However, the adsorbent was able to absorb a good amount of methylene blue. The diagram of ∆PH–initial pH of magnetic nanobiocomposite is shown in Fig. 7.

### The effect of time on the absorption process

**Figure 8:**
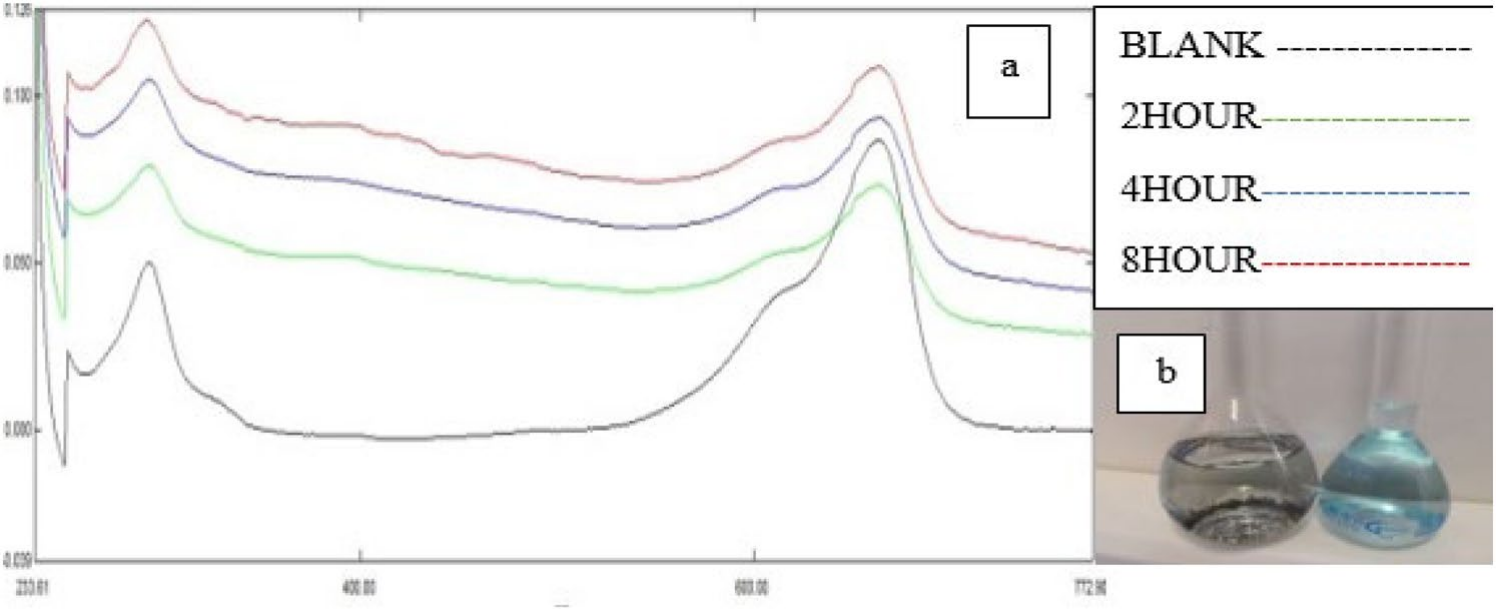
(**a**) UV–Vis spectra of the Methylene blue solutions at stop time 2, 4, 8 and the control sample (**b**) Comparison of the control sample and the absorbed sample after 8 h.

UV–Vis spectra of the Methylene blue solutions at stop time 2, 4, 8 and the control sample is shown in Fig. 8a and Comparison of the control sample and the absorbed sample after 8 h is shown in Fig. 8b. As shown in Fig. 8a, with time, the amount of adsorbed dye increases because the depth of the peaks is less than that of the control sample. And as shown in Fig. 8b, finally after 8 h, the maximum amount of dye is absorbed, which is the final concentration of the solution is 6 mg/L, the amount of dye absorbed after 8 h is q8 = 388 mg/g and the amount of color removal was determined to be 97%.

## Conclusion

Ni_0.5_Co_0.5_Fe_2_O_4_/AC@Ch nanoparticles were synthesized through co-precipitation and ultrasonic waves and used as a magnetic nanobiocomposite to remove pollutants. Based on BET analysis, the surface area of the nanobiocomposite was 316 m^2^/g. The methylene blue absorption test showed an absorption rate of more than 97% after 8 h. According to the FESEM images, the particle size was about 17 nm, and FTIR and EDAX analysis showed that this compound had a purity of 99% and the reaction was successful. The cubic spinel structure of nickel–cobalt ferrite and the successful coating of chitosan on the surface of the nano-absorbent were confirmed through XRD analysis, and the size of the crystals at the wavelength of 96 nm was obtained through the Bragg equation. FT-IR spectrum of nanoparticles after adsorption confirmed the presence of methylene blue on the nanobiocomposite surface. Ni_0.5_Co_0.5_Fe_2_O_4_/AC@Ch can be used as a biocompatible adsorbent due to its large specific surface area and high reactivity, and it is easily removed from aqueous solutions by magnetic separation method.

## Data Availability

The datasets used and analaysed during the current study available from the corresponding author on reasonable request.
